# The Colocalization Potential of HIV-Specific CD8^+^ and CD4^+^ T-Cells is Mediated by Integrin β7 but Not CCR6 and Regulated by Retinoic Acid

**DOI:** 10.1371/journal.pone.0032964

**Published:** 2012-03-28

**Authors:** Vanessa Sue Wacleche, Nicolas Chomont, Annie Gosselin, Patricia Monteiro, Mathieu Goupil, Hassen Kared, Cécile Tremblay, Nicole Bernard, Mohamed-Rachid Boulassel, Jean-Pierre Routy, Petronela Ancuta

**Affiliations:** 1 Department of Microbiology and Immunology, Université de Montréal, Montreal, Quebec, Canada; 2 Centre Hospitalier de l'Université de Montréal (CHUM)-Research Center, Saint-Luc Hospital, Montreal, Quebec, Canada; 3 VGTI-Florida, Port St Lucie, Florida, United States of America; 4 INSERM Unit 743, Montréal, Quebec, Canada; 5 Research Institute of the McGill University Health Centre, Montreal, Quebec, Canada; 6 Division of Hematology, McGill University Health Centre, Montreal, Quebec, Canada; 7 Immunodeficiency Service, Montreal Chest Institute, McGill University Health Centre, Montreal, Quebec, Canada; South Texas Veterans Health Care System and University Health Science Center San Antonio, United States of America

## Abstract

CD4^+^ T-cells from gut-associated lymphoid tissues (GALT) are major targets for HIV-1 infection. Recruitment of excess effector CD8^+^ T-cells in the proximity of target cells is critical for the control of viral replication. Here, we investigated the colocalization potential of HIV-specific CD8^+^ and CD4^+^ T-cells into the GALT and explored the role of retinoic acid (RA) in regulating this process in a cohort of HIV-infected subjects with slow disease progression. The expression of the gut-homing molecules integrin β7, CCR6, and CXCR3 was identified as a “signature” for HIV-specific but not CMV-specific CD4^+^ T-cells thus providing a new explanation for their enhanced permissiveness to infection *in vivo*. HIV-specific CD8^+^ T-cells also expressed high levels of integrin β7 and CXCR3; however CCR6 was detected at superior levels on HIV-specific CD4^+^ *versus* CD8^+^ T-cells. All trans RA (ATRA) upregulated the expression of integrin β7 but not CCR6 on HIV-specific T-cells. Together, these results suggest that HIV-specific CD8^+^ T-cells may colocalize in excess with CD4^+^ T-cells into the GALT *via* integrin β7 and CXCR3, but not *via* CCR6. Considering our previous findings that CCR6^+^CD4^+^ T-cells are major cellular targets for HIV-DNA integration *in vivo*, a limited ability of CD8^+^ T-cells to migrate in the vicinity of CCR6^+^CD4^+^ T-cells may facilitate HIV replication and dissemination at mucosal sites.

## Introduction

The human immunodeficiency virus type 1 (HIV) epidemic remains a major global health problem despite major advances made since the discovery of the virus in 1983 [1]. The HIV infection has been known to be associated to a gastrointestinal pathology since the beginning of the epidemic [2], [3]. Recent studies in HIV-infected individuals and simian models of infection demonstrated that depletion of CD4^+^ T-cells from gut-associated lymphoid tissues (GALT) occurs very early upon infection [4], [5], [6], [7]. Memory CD4^+^ T-cells, expressing the HIV coreceptor CCR5, massively infiltrate the GALT and are preferential targets of viral replication and depletion [8], [9]. The alteration of GALT homeostasis in HIV-infected individuals leads to the impairment of mucosal immunity and microbial translocation from the gut, which can drive chronic immune activation [10], [11]. Despite a partial restoration of mucosal immunity in the GALT of individuals receiving long-term antiretroviral therapies (ART) [12], [13], viral reservoirs persist in different cellular and anatomic compartments and represent a major barrier to HIV eradication [14].

A small fraction of HIV-infected individuals control disease progression for a long period of time in the absence of ART and are called long-term nonprogressors (LTNPs) or slow progressors [15]. The LTNPs subjects are generally defined as HIV-infected individuals who remain clinically asymptomatic with non-declining CD4 counts (>500 cells/µl), and undetectable plasma viral loads (<50–75 HIV RNA copies/ml) for >10 years [16]. However, LTNPs are very heterogeneous and include individuals with virological and/or immunological control. Virological controllers (also called “elite controllers”) are characterized by undetectable plasma viral loads regardless of their CD4 counts, while immunological controllers maintain their CD4 counts in the normal range despite detectable plasma viral loads [15], [16]. Mechanisms involved in the control of disease progression in LTNPs have been linked to host genetic factors controlling the quality of innate and adaptive immunity [17], [18]. The ability of CD8^+^ T-cells to control HIV replication *via* cytotoxic and non-cytotoxic mechanisms is well documented [19], [20], [21]. However, viral reservoirs persist in LTNPs [22], [23], pointing out the inability of the immune system to achieve HIV eradication. This is consistent with the finding that the GALT remains an important target of HIV replication in LTNPs with functional alterations in this compartment contributing to slow disease progression [24]. Nevertheless, the existence of a group of HIV-exposed uninfected individuals, in which HIV-specific CD8^+^ T-cell responses were detected in the cervical mucosa [25], provides proof that protective immunity against HIV can be mounted under specific conditions. Thus, the mechanisms of immune protection against HIV require further investigations.

HIV infection is initiated by a small viral founder population that undergoes mutations to escape T-cell responses [26], [27], [28]. Limiting viral dissemination from the portal site of entry very early after infection *via* robust anti-viral mechanisms is of paramount importance to prevent the establishment of a chronic HIV infection [28]. Recent studies using a model of simian immunodeficiency virus (SIV)-infection and *in situ* visualization techniques demonstrated that SIV-specific CD8^+^ T-cells (effectors) are recruited into the vaginal mucosa and lymph nodes in close proximity to SIV-infected CD4^+^ T-cells (targets) [29]. The spatial proximity of excess effectors *versus* target cells appears to be critical for the control of SIV replication and dissemination *in vivo* [29]. By analogy, the colocalization potential of HIV-specific CD8^+^ and CD4^+^ T-cells into tissues such as the GALT might determine the extent of viral dissemination and the outcome of disease progression.

Trafficking of peripheral blood T-cells into the GALT is mediated *via* specific adhesion molecules and chemokine receptors. The integrin α4β7 binds to the mucosal addressin cell adhesion molecule-1 (MadCAM-1) expressed on gut endothelial cells and allows cells to cross the endothelial barrier [30]. The integrin αEβ7 binds to the E-cadherin expressed on the basolateral surface of intestinal epithelial cells and contributes to cell retention in the intraepithelial compartment [31]. The CCR6 is important in the recruitment of T-cells into Peyer's Patches [32], [33], [34], while CCR9 mediates T-cell infiltration into lamina propria [35], [36], [37]. The CCR5 and CXCR3 binding chemokines also regulate infiltration of T-cells into the gut [38], [39]. Previous studies reported the expression of gut-homing molecules on HIV-specific CD8^+^ or CD4^+^ T-cells. The HIV-specific CD4^+^ T-cells express the integrin β7 and CCR5 [40], [41], while HIV-specific CD8^+^ T-cells from the gut express CCR5 and integrin αEβ7 [42]. In addition, a fraction of HIV-specific CD4^+^ and CD8^+^ T-cells express CCR6 [43].

Results from our group and those published by others identified CCR6 as a marker for memory CD4^+^ T-cells that are highly permissive to HIV infection *in vitro* [44], [45] and carry superior levels of integrated HIV-DNA *in vivo* [44], [45]. Also, we demonstrated that treatment with retinoic acid (RA), a metabolite of vitamin A responsible for the imprinting for gut homing [46], [47], significantly increased the permissiveness of CCR6^+^ but not CCR6^−^ CD4^+^ T-cells to HIV replication by acting at entry (CCR5 upregulation) and yet unidentified post-entry levels [48]. Thus, CCR6^+^CD4^+^ T-cells may represent sites for active HIV replication into the GALT. The ability of HIV-specific CD8^+^ T-cells to be recruited into the GALT in the vicinity of CCR6^+^CD4^+^ T-cells remains unknown and might be predictive of an efficient control of HIV replication in target cells at the portal sites of entry.

In this study, we investigated the potential of total and HIV-specific CD8^+^ T-cells to colocalize in excess with CCR6^+^CD4^+^ T-cells and explored the role of the RA pathway in regulating the gut-homing potential of these cells. We report here a decreased frequency of CD8^+^ and CD4^+^ T-cells expressing CCR6 in the peripheral blood of HIV-infected subjects regardless of their clinical characteristics of disease progression. In a cohort of HIV-infected subjects with slow disease progression, HIV-specific *versus* CMV-specific CD4^+^ T-cells highly express the gut-homing markers integrin β7, CCR6, and CXCR3, suggesting a link between enhanced permissiveness to infection in HIV-specific CD4^+^ T-cells [49] and their gut-homing potential. HIV-specific CD8^+^ T-cells also express the gut-homing molecules integrin β7 and CXCR3 but express low levels of CCR6. Thus, HIV-specific CD8^+^ T-cells may migrate into the gut *via* integrin β7 and CXCR3 but exhibit a limited potential to colocalize with CD4^+^ T-cell in certain GALT sites where recruitment is dependent on CCR6 (*e.g.*, Peyer's Patches) [32], [33], [34]. This is consistent with our previous finding that CCR6^+^CD4^+^ T-cells are major sites for HIV-DNA integration *in vivo* [44]. Together these results suggest that, in addition to other previously described cellular features (*e.g.*, antiviral properties, poly-functionality, and exhaustion), the co-localization potential of HIV-specific CD4^+^ and CD8^+^ T-cells might represent a new parameter to consider in order to predict the efficacy of anti-HIV responses. Future therapeutic strategies should aim at increasing the colocalization potential of HIV-specific effector and target cells in mucosal tissues for a better control of HIV dissemination from the portal sites of entry.

## Materials and Methods

### Study subjects

Included in the study were three groups of HIV-infected subjects: (i) treatment-naïve with slow disease progression (SP, slow progressors; n = 14), (ii) recently infected untreated (RI; n = 18), and chronically infected under viral-suppressive anti-retroviral therapy (CI on ART; n = 20). A cohort of n = 13 HIV-uninfected subjects were included in this study as controls. Tables 1, 2, and 3 contain information on the CD4 and CD8 counts, plasma viral loads, and time since infection of SP, RI, and CI on ART HIV-infected subjects, respectively. At the time of leukapheresis, 9/14 SP subjects satisfied the long-term nonprogressor (LTNP) criteria, which include >7 years time since infection, low to undetectable plasma viral load in the absence of ART, and CD4 counts >500 cells/µl [15], [16], while 5/14 SP subjects had CD4 counts <500 cells/µl (Table 1). Among the later group, three subjects (SP 005, 011, 0108) were infected for >19-years and had detectable plasma viral loads, while two subjects (SP 015, 0102) had undetectable plasma viral loads and time since infection >9-years (Table 1). SP subjects with CD4 counts <500 cells/µl were not treated as they lacked clinical signs of immunological failure and maintained their plasma viral load <10^4^ HIV RNA copies/ml. CI on ART subjects received various antiviral regimens containing a protease inhibitor, a non-nucleoside reverse transcriptase inhibitor, or three nucleoside reverse transcriptase inhibitors, as we previously reported [44]. Plasma viral load was measured using the Amplicor HIV-1 monitor ultrasensitive method (Roche). PBMCs (10^9^–10^10^ cells) were collected from HIV-infected and uninfected subjects by leukapheresis, as previously reported [50]. HIV-subjects were recruited *via* the HIV Primo Infection cohort at the McGill University Health Centre, Royal Victoria Hospital, Montreal, or from the Canadian Cohort of HIV+ Slow Progressors.

**Table 1:** Clinical parameters of HIV-infected subjects with slow disease progression (SP).

Subjects	CD4 counts#	CD8 counts#	Plasma viral load&	Time since infection*	ART
**SP 001**	860	1148	158	15	No
**SP 005**	435	694	1862	19	No
**SP 006**	991	921	<50	15	No
**SP 007**	720	631	<50	19	No
**SP 008**	670	475	<50	15	No
**SP 011**	325	941	5370	19	No
**SP 015**	448	403	<50	16	No
**SP 0101**	1080	1320	<50	11	No
**SP 0102**	440	162	<50	9	No
**SP 0105**	780	1020	15920	12	No
**SP 0106**	520	1790	86128	12	No
**SP 0107**	720	2840	1769	18	No
**SP 0108**	387	465	2937	21	No
**SP 0109**	670	740	1942	19	No
**Median**	**670**	**831**	**964**	**16**	

# , cells/µl;

& , HIV RNA copies per ml plasma (log_10_);

* , years; ART, antiretroviral therapy.

**Table 2:** Clinical parameters of recently HIV-infected (RI) untreated subjects.

Subjects	CD4 counts#	CD8 counts#	Plasma viral load&	Time since infection*	ART
**RI 001**	704	1081	68412	9	No
**RI 002**	310	350	200363	46	No
**RI 003**	522	366	2021	5	No
**RI 004**	691	1122	8714	12	No
**RI 005**	341	372	16883	5	No
**RI 006**	483	930	366646	2	No
**RI 007**	857	1499	93223	3	No
**RI 008**	475	640	56838	2	No
**RI 009**	338	1829	81984	4	No
**RI 010**	378	779	93706	2	No
**RI 011**	443	736	176557	5	No
**RI 012**	442	538	36349	5	No
**RI 013**	571	1266	5897	7	No
**RI 014**	824	626	1167770	6	No
**RI 015**	494	1055	15703	16	No
**RI 016**	730	1310	97044	25	No
**RI 017**	255	988	52835	25	No
**RI 018**	316	376	57154	8	No
**Median**	**479**	**855**	**62,783**	**5.7**	

# , cells/µl;

& , HIV RNA copies per ml plasma (log_10_);

* , months; ART, antiretroviral therapy.

**Table 3:** Clinical parameters of chronically HIV-infected subjects under long-term viral suppressive ART (CI on ART).

Subjects	CD4 counts#	CD8 counts#	Plasma viral load&	Time since infection*	ART
**CI 001**	890	673	<50	57	Yes
**CI 002**	463	757	<50	152	Yes
**CI 003**	602	767	<50	158	Yes
**CI 004**	563	613	<50	86	Yes
**CI 005**	424	461	<50	84	Yes
**CI 006**	731	413	<50	51	Yes
**CI 007**	834	527	<50	38	Yes
**CI 008**	552	715	<50	139	Yes
**CI 009**	671	1120	<50	242	Yes
**CI 010**	510	765	<50	61	Yes
**CI 011**	799	1727	<50	62	Yes
**CI 012**	501	278	<50	90	Yes
**CI 013**	344	642	<50	59	Yes
**CI 014**	604	1281	<50	53	Yes
**CI 015**	443	322	<50	18	Yes
**CI 016**	599	923	<50	86	Yes
**CI 017**	688	1273	<50	100	Yes
**CI 018**	434	583	<50	165	Yes
**CI 019**	492	582	<50	170	Yes
**CI 020**	529	690	<50	49	Yes
**Median**	**558**	**682**	**<50**	**85**	

# , cells/µl;

& , HIV RNA copies per ml plasma (log_10_);

* , months; ART, antiretroviral therapy.

### Ethics statement

This study using PBMC samples from HIV-infected and uninfected subjects, was conducted in compliance with the principles included in the Declaration of Helsinki. This study received approval from the Institution Review Board of the McGill University Health Center and CHUM-Research Center, Montreal, Canada. All blood donors provided written informed consent for their participation to the study.

### Antibodies and polychromatic flow cytometry analysis

Fluorochrome-conjugated Abs used for polychromatic flow cytometry analysis were CD3-Pacific Blue (UCHT1), CD3-PE/Cy7 (SK7) CD4-Alexa700 (RPA-T4), CD8 APC-Cy7 (SKI), CCR4-PE/Cy7 (1G1), CXCR3-PE/Cy5 (1C6), CD154-PE/Cy5 (89-76), CCR5-PE (2D7) β7-PE/Cy5 (FIB504), CCR6-PE (11A9) and IFN-γ-Alexa700 (B27) (BD Biosciences); CXCR3-FITC (49801) (R&D Systems); β7-PE (FIB504) and IL-17A-PE (ebio64DEC17) and TNF-α-Pacific Blue (MAB11) (eBioscience). The β7 chain may associate with the α4 chain to form the α4β7 integrin or with the αE chain to form the αEβ7 integrin. In previous studies, we demonstrated that the majority of peripheral blood T cells expressed α4 but not the αE chain [48]. Based on this evidence, we can assume the antibody against the β7 chain used in our study identifies the α4β7 dimer.

For extracellular staining, PBMCs were washed with FACS buffer (PBS 1X, 10% FBS (v/v) (Sigma), 0.02% sodium azide (weight/volume)), stained with specific antibodies for 20 minutes at 4°C, washed with FACS buffer, and fixed with a 2% paraformaldehyde buffer. For cell phenotype analysis of antigen-specific T-cells by flow cytometry, 200–5,000 events were acquired using a BD LSRII flow cytometer. A viability stain (Vivid, Invitrogen) was included in the specific staining cocktails to exclude dead cells from our analysis. Results were analyzed using the BD Diva software. Prior to use, all Abs were titrated for an optimal signal to noise ratio. All Abs cocktails were validated by comparing single to multiple staining, and gates were established using fluorescence minus one (FMO), as previously described [44].

### HIV-1 peptide pool preparation

Stimulatory peptides were 15-mers with 11 amino acid overlaps corresponding to HIV-1 clade B consensus Gag (n = 123), Nef (n = 49), and Pol (n = 249) (National Institute of Health (NIH) AIDS Research and Reference Reagent Program, Germantown, MD). Each peptide was diluted in DMSO at 12.5–50 mg peptide/ml, depending on the peptide solubility and stored at −80°C. These were used for the preparation of peptide pools (50–100 µg peptide/ml), containing 11 to 28 peptides per pool, as described in Table 4 and Table S1. Pools equivalent to the complete sequence of Nef (100 µg peptide/ml), Gag (100 µg peptide/ml), and Pol (50 µg peptide/ml) proteins were also prepared. Peptide pools were stored at −80°C and used for CD154 assays (10 µg peptide/ml) and proliferation assays (500 ng peptide/ml)

**Table 4:** Screening for HIV-1 specific CD4^+^ and CD8^+^ T-cells responses using the cell proliferation CFSE dilution assay.

	% CFSE^low^ T-cells									
Subjects	SP 001		SP 006		SP 007		SP 011		SP 015	
T-cells	CD4^+^	CD8^+^	CD4^+^	CD8^+^	CD4^+^	CD8^+^	CD4^+^	CD8^+^	CD4^+^	CD8^+^
Medium#	0.06	0.12	0.12	0.21	0.13	0.1	0.14	0.13	0.11	0.15
SEB	39.8&	49.9	21.5	39.01	40.76	42.07	37.36	42.57	53.01	55.27
CMV pp65	1.05	2.3	0.36	3.9	57.69	8.94	2.32	3.71	25.58	25.99
HIV p24	-#	-	-	-	1.7	1.4	-	-	0.74	1.17
Nef _5139–5187_	0.23	0.57	-	-	-	2.87	-	0.36	0.27	0.69
Nef _5139–5163_	0.2	0.32	-	-	-	2.1	-	-	-	0.44
Nef _5164–5187_	-	0.34	-	-	-	0.52	-	-	-	-
Gag _705–827_	0.5	5.55	0.55	1.28	1.38	13.92	-	1.26	1.1	3.03
Gag _705–728_	0.31	1.18	-	-	0.29	0.5	-	1.14	-	-
Gag _729–752_	-	-	-	-	-	2.91	-	-	0.28	-
Gag _753–776_	0.21	2.29	-	-	1.73	1.48	-	-	-	-
Gag _777–800_	0.24	2.33	-	-	0.61	19.53	-	0.4	0.86	2.19
Gag _801–827_	-	-	-	-	-	-	-	-	0.74	0.78
Pol _461–709_	0.54	4.13	0.25	0.89	0.38	6.38	-	1.13	0.25	2.26
Pol _461–484_	-	-	-	-	-	0.32	-	-	-	-
Pol _485–508_	0.19	-	-	-	-	-	-	-	0.45	0.4
Pol _509–532_	-	-	-	-	-	-	-	-	-	-
Pol _533–556_	0.22	0.23	-	-	-	-	-	-	0.29	4.75
Pol _557–580_	-	-	-	-	-	3.89	-	-	-	0.38
Pol _581–604_	-	-	-	-	-	-	-	-	-	0.5
Pol _605–628_	0.39	0.39	0.3	-	-	0.29	-	-	-	1.28
Pol _629–652_	0.45	0.47	-	-	3.79	0.24	-	-	-	0.73
Pol _653–674_	0.54	0.47	-	-	-	0.21	-	-	-	0.31
Pol _675–698_	0.44	2.58	0.24	-	-	0.22	-	-	0.32	0.89
Pol _699–709_	0.42	0.37	-	-	-	-	-	-	-	-
Nef Gag Pol	1.87	9.21	0.34	1.01	1.47	14.05	0.56	1.92	0.82	3.65

# , background proliferation;

& , T-cell proliferation was considered positive when the % of CFSE^low^ T-cells in antigen-stimulated compared to the background was >2-fold higher.

### *Candida albicans hyphaes* culture and lysis

*Candida albicans hyphaes* was used a s positive control for the induction of IL-17 production by T-cells (Figure S1), as previously described [51]. *Candida albicans* LAM-1 strain was provided by Dr. Louis de Repentigny (University of Montreal, Montreal, Quebec, Canada), as colonies in a Petri dish. From the isolated colonies, the yeast form was cultured overnight at 37°C in Yeast Peptone Dextrose (YPD) medium (BD Bioscience). To induce the transition from the yeasts form to hyphaes, 0.1–0.5×10^6^ yeasts/ml were cultured in YPD media 20% FBS and incubated for 4 hours at 37°C. The hyphaes generated were washed and resuspended at 2×10^6^ cellules/100 µl in PBS (GIBCO). Micro glass-beads (SIGMA) were added, and cell lysis was performed using the FastPrep FP120 instrument (Thermo Savant, Carlsbad, CA). Cells were lyzed at a speed of 5 meter/second 4 times for 30 seconds and then placed on ice for 2 minutes; this step was repeated 10 times. Lysates were stored frozen at −20°C. Proliferation assays were performed to determine the optimal immunogenic concentrations of *Candida albicans hyphae*.

### CD154/CD40L assay

To identify antigen-specific CD4^+^ T-cells, the CD154/CD40L assay was performed as previously described (89). Briefly, PBMC from HIV-infected subjects were resuspended in RPMI 1640 (GIBCO), 100 units/ml Penicillin (GIBCO), 100 µg/ml Streptomycin (GIBCO), and 2 mM of L-glutamine (RPMI) with 10% FBS (SIGMA) at 10×10^6^ cells/ml. Cell suspension (200 µl/well) were plated into 96-well plates and stimulated with 1 µg/ml *Staphylococcal enterotoxin B* (SEB) (Toxin Technology), 5 µg/ml of *Cytomegalovirus* (CMV) pp65 peptide pool (Miltenyi), 5 µg/ml recombinant HIV-p24 protein (ImmunoDiagnostics, Inc.), or 10 µg/ml of HIV peptide pools (NIAID AIDS Reagent Program) in the presence of 20 µl/well of anti-CD154-PE/Cy5 Abs (BD Biosciences) and 2 µM of monensin (SIGMA) for 16 hrs at 37°C. Cells were then harvested, stained for surface markers with fluorescence-conjugated Abs against CD3, CD4, integrin β7, CCR6, CXCR3, and CCR4, and analyzed by flow cytometry for the expression of homing markers on CD3^+^CD4^+^CD154^+^ T-cells.

### CFSE dilution assay

To detect antigen-specific T-cell proliferation, the Carboxy Fluoroscein Succinimidyl Ester (CFSE) dilution assay was performed as previously described [52]. Briefly, PBMC were loaded with 0.5 µM CFSE (Sigma) for 8 minutes at room temperature. The optimal concentration for CFSE was determined by titration for each CFSE lot. Cells were then washed once with PBS and once with RPMI 1640, and then cultured in 5 ml polypropylene tubes (Becton Dickinson) at 2×10^6^ cells/ml in RPMI with 10% human serum (Gemini). Cells were stimulated with HIV peptide pools in which each peptide was at a concentration of 500 ng/ml, 5 µg/ml recombinant HIV-p24 protein (ImmunoDiagnostics), 25 ng/ml SEB (Toxin Technologies) or 1 µg/ml pp65 CMV peptide pool (Miltenyi) for 6 days at 37°C. Cells were harvested, stained with fluorescence-conjugated Abs against CD3, CD4, integrin β7, CCR6, CXCR3, CCR4, and/or CCR5, and analyzed by flow cytometry for the phenotype of CD3^+^CD4^+^CFSE^low^ and CD3^+^CD4^−^CFSE^low^ cells. In preliminary experiments, we demonstrated that the majority (>95%) of CD3^+^CD4^−^ cells were CD8^+^ T-cells. When indicated, a viability stain (Vivid; Invitrogen) was included in staining cocktails to exclude dead cells from analysis.

### Intracellular staining for cytokines

CFSE loaded PBMC were stimulated with antigen for 5 days and then restimulated with 50 ng/ml PMA (SIGMA) and 1 µg/ml Ionomycin (SIGMA) in the presence of 10 µg/ml Brefeldin A (SIGMA) for 18 hours. The production of IFN-γ, TNF-α, and IL-17A was measured by intracellular staining with appropriate Abs using the BD Cytofix/Cytoperm kit (BD Biosciences) according to the manufacturer's protocol.

### Statistics

The significance of differences observed between-groups was assessed using Mann-Whitney tests (for unpaired samples) and Paired t-test (for paired samples) as specified in figure legends. The correlation between study variables was assessed using a Spearman correlation test and linear regression models. All statistical analyses were performed using the GraphPad Prism 5 software. P-values <0.05 were considered significant.

## Results

**Decreased frequency of CCR6-expressing CD8^+^ and CD4^+^ T-cells in the peripheral blood of HIV-infected subjects with slow and rapid disease progression:** We previously identified CCR6 as a marker for memory CD4^+^ T-cells being highly permissive to HIV infection *in vitro* and major sites for HIV-DNA integration in infected subjects [44], [48]. As a consequence, the frequency of circulating CCR6^+^CD4^+^ T-cells is dramatically reduced from the early stages of HIV infection and the normalization of this frequency is not observed under viral suppressive ART [44]. Considering the antiviral properties of CD8^+^ T-cells [19], [53], we hypothesized that a robust control of HIV disease progression is dependent on the ability of CD8^+^ T-cells to co-localize with CCR6-expressing CD4^+^ T-cells. To test this hypothesis, the expression of CCR6 was first quantified on peripheral blood CD8^+^ and CD4^+^ T-cells from HIV-infected subjects with slow and rapid disease progression. The cohort of slow progressors (SP; n = 14) included HIV-infected subjects with a median time since infection of 16 years, median CD4 counts of 670 cells/µl, median CD4 counts of 831 cells/µl, and undetectable or low plasma viral loads (median: 964 HIV RNA copies/ml) in the absence of antiretroviral therapy (ART) (Table 1). The cohort of HIV-infected progressors included recently infected untreated (RI; n = 18; median CD4 counts: 479 cells/µl; median CD8 counts: 855 cells/µl; median plasma viral load: 62,783 HIV RNA copies/ml; median time since infection: 5.7 months) (Table 2) and chronically infected under long-term (>1-year) viral-suppressive ART (CI on ART; n = 20; median CD4 counts: 558 cells/µl; median CD8 counts: 682 cells/µl; median plasma viral load: <50 HIV RNA copies/ml; median time since infection: 85 months) subjects (Table 3). The frequency of CCR6-expressing CD8^+^ and CD4^+^ T-cells and the CD8/CD4 ratios within total and CCR6^+^ T-cell fractions were compared between HIV-uninfected and the three groups of HIV-infected subjects.

**Figure 1:**
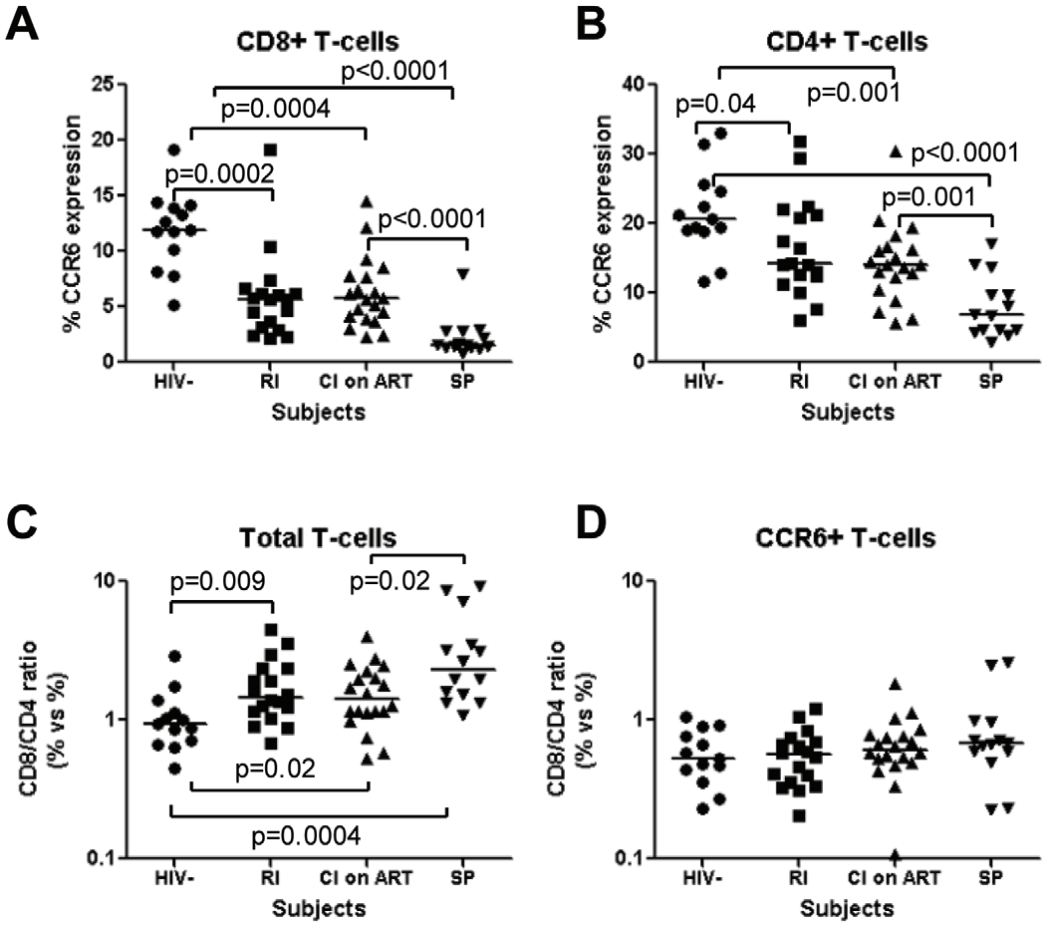
The frequency of CD8^+^ and CD4^+^ T-cells expressing CCR6 is decreased in HIV infected subjects with slow and rapid disease progression. Peripheral blood mononuclear cells (PBMC) from HIV-uninfected (HIV-; n = 13) and HIV+ subjects recently infected untreated (RI, n = 18), chronically infected under long-term viral suppressive antiretroviral therapy (CI on ART; n = 20), and slow progressors (SP, n = 14) were stained with a cocktail of fluorescence-conjugated CD3, CD4, and CCR6 Abs. The frequency of (**A**) CD3^+^CD4^−^ T-cells (referred to as CD8^+^ T-cells) and (**B**) CD3^+^CD4^+^ T-cells (referred to as CD4^+^ T-cells) expressing CCR6 was quantified by polychromatic flow cytometry and compared between HIV-uninfected controls and RI, CI on ART, and SP HIV-infected subjects. The CD8/CD4 T-cell ratios were calculated within the total (**C**) and CCR6^+^ T-cell fractions (**D**) in the group of HIV-uninfected controls and the three groups of HIV-infected subjects. Horizontal bars indicate median values. Mann-Whitney p-values are indicated in the figures.

The frequency of CCR6-expressing CD8^+^ and CD4^+^ T-cells was significantly decreased in RI and CI on ART subjects compared to uninfected controls; unexpectedly, this frequency was also significantly decreased in SP compared to uninfected controls and CI on ART subjects (Figure 1A–B). The CD8/CD4 ratios within the total T-cell population were significantly higher in RI (median: 1.4), CI on ART (median: 1.4), and SP subjects (median: 2.3) compared to uninfected controls (median: 0.9) and also in SP compared to CI on ART subjects (Figure 1C). This suggests the potential recruitment of excess CD8^+^ T-cells in the vicinity of CD4^+^ T-cells. In contrast, the median CD8/CD4 ratios within the CCR6^+^ fraction were <1 in HIV-infected and uninfected subjects, with no significant differences between RI, CI on ART, and SP subjects (Figure 1D). No significant correlations were found between CD4 counts or plasma viral loads and all four parameters investigated in Figure 1 within the three HIV-infected groups (data not shown). These results demonstrate an alteration in the frequency of CCR6-expressing CD8^+^ and CD4^+^ T-cells in HIV-infected subjects regardless of their clinical characteristics of disease progression. These results suggest the inability of CD8^+^ T-cells to be recruited in excess in the proximity of CCR6^+^CD4^+^ T-cells, and this even in subjects with slow disease progression.

**CD4^+^ T-cells specific for HIV** ***versus*** **CMV preferentially express gut-homing markers:** HIV preferentially infects HIV-specific CD4^+^ T-cells, while CMV-specific CD4^+^ T-cells are relatively resistant to infection *in vivo* [41], [49], [54]. This coincides with the fact that HIV-specific CD4^+^ T-cells express higher levels of the HIV CCR5 coreceptor and produce lower levels of CCR5 binding chemokines than do CMV-specific CD4^+^ T-cells [41], [49], [54]. We hypothesized that differences in viral permissiveness between HIV-specific *versus* CMV-specific CD4^+^ T-cells are also related to their distinct ability to home into anatomic sites of active viral replication, such as the GALT. To test this hypothesis, we investigated the gut-homing potential on CD4^+^ T-cells specific for HIV *versus* CMV. The SEB superantigen is known to induce polyclonal T-cell activation [52] and was used as a positive control. The tissue-specific homing molecules studied were integrin β7 for the migration across the GALT endothelium [30], [39], [46], CCR6 for the migration into the GALT Peyer's Patches [33], [55], CXCR3 for the migration into inflammatory sites, including the GALT [30], [39], [46], [56], and CCR4 for the migration into the skin [57]. Experiments were performed with PBMC from seven HIV-infected treatment-naïve subjects with slow disease progression (Table 1), because they exhibited relatively high frequencies of HIV-specific CD4^+^ and CD8^+^ T-cells (Table 4 and Table S1). This choice is also justified by the fact that the frequency of CD4^+^ and CD8^+^ T-cells expressing CCR6 is also altered in the peripheral blood of SP subjects compared to uninfected controls (Figure 1).

In a first experimental approach, antigen-specific CD4^+^ T-cells were identified based on their expression of CD154 (CD40 ligand, CD40L) using flow cytometry analysis (Figure S1A), as previously described by others [58]. The PBMCs from HIV-infected individuals were screened for the ability to respond to an antigen panel that included HIV Nef (n = 3), Gag (n = 6) and Pol (n = 11) overlapping peptide pools, HIV-p24 recombinant protein, SEB, and CMV-pp65 recombinant protein (Table S1). The PBMCs were then stimulated with the most immunogenic antigenic panel and the expression of integrin β7, CCR6, CXCR3, and CCR4 was analyzed on antigen-specific CD154^+^CD4^+^ T-cells by polychromatic flow cytometry (Figure S1A–B). The phenotype of CD154^+^CD4^+^ T-cells specific for different HIV peptide pools was highly heterogeneous within the same donor. Also, inter-donor variations were observed in the expression of homing molecules on CD154^+^CD4^+^ T-cells, even those specific for the same HIV peptide pool (Figure S1C). Regardless of this heterogeneity, statistical analysis of homing molecule expression demonstrated that HIV-specific compared to CMV-specific CD154^+^CD4^+^ T-cells expressed significantly higher levels of the integrin β7 (Figure S1D). In addition, HIV-specific compared to SEB-specific T-cells displayed increased expression of the integrin β7, CCR6, CXCR3, and CCR4 (Figure S1D). These results demonstrate that HIV-specific CD154^+^CD4^+^ T-cells distinguish from cells of other antigenic specificities (CMV, SEB) by their high expression of both gut-homing markers integrin β7 and CCR6.

**Figure 2 pone-0032964-g002:**
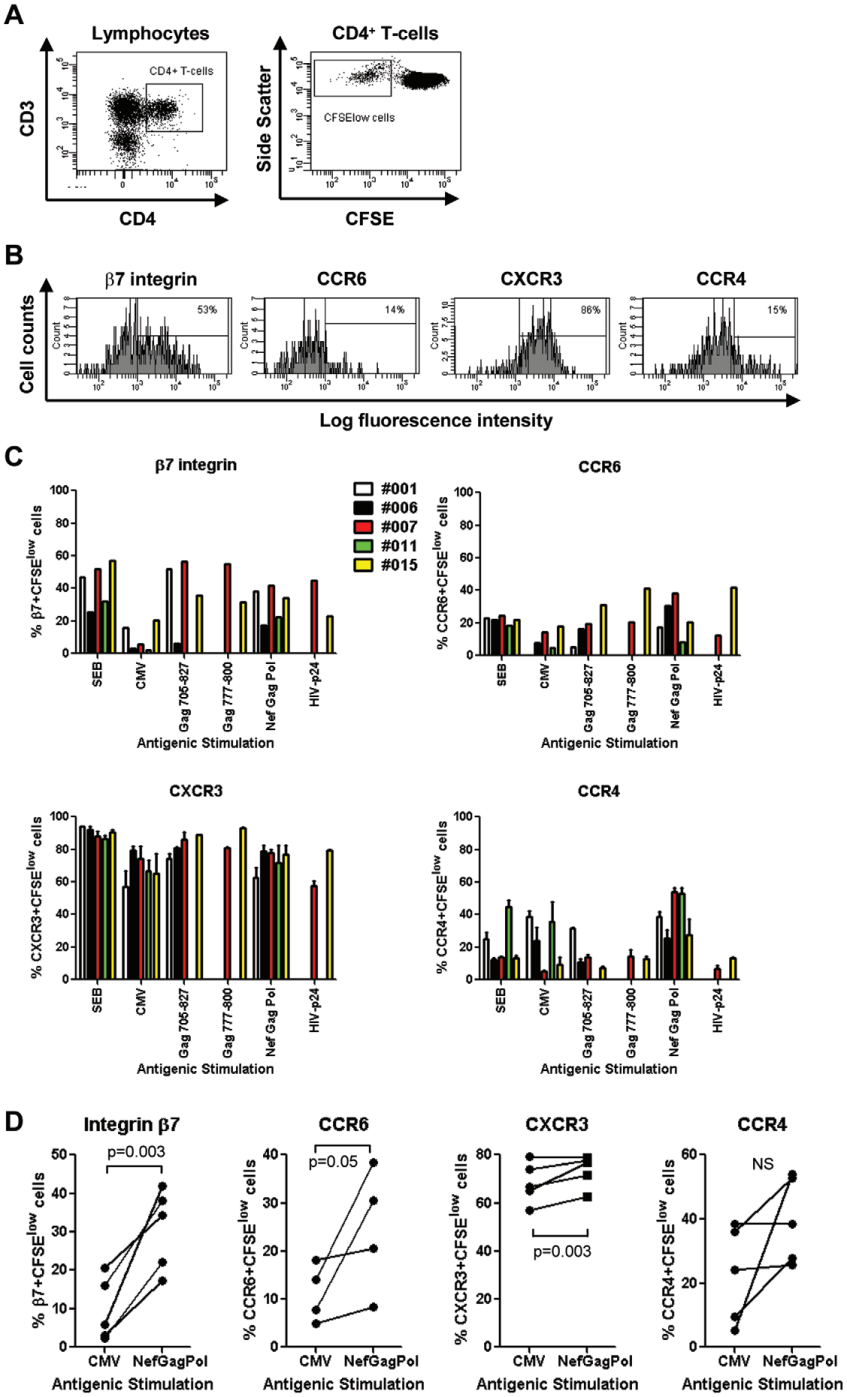
Preferential gut-homing potential of HIV-specific *versus* CMV-specific CD4^+^ T-cells. PBMC from SP subjects were loaded in CFSE (0.5 µM) and stimulated with different HIV Nef, Gag, Pol peptide pools (500 ng/ml), recombinant HIV-p24 (5 µg/ml), SEB (25 ng/ml), or the recombinant CMV-pp65 peptide pool (1 µg/ml) for 6 days at 37°C. Antigen-specific T-cells were identified as CFSE^low^ cells, as previously described [52]. Cells were stained with a cocktail of fluorescence-conjugated CD3, CD4, integrin β7, CCR6, CXCR3, and CCR4 Abs and analyzed by polychromatic flow cytometry for (**A**) the frequency of CFSE^low^CD3^+^CD4^+^ T-cells (referred to as CD4^+^ T-cells) and (**B–D**) the expression of integrin β7, CCR6, CXCR3, and CCR4 on antigen-specific CFSE^low^CD4^+^ T-cells. (**A–B**) Shown are results from one donor (i.e., SP 007) generated upon stimulation of PBMC with HIV Gag_705–827_ peptide pool, representative of results generated with cells from five different donors. (**C**) Shown is the expression of the homing receptors on CFSE^low^CD4^+^ T-cells specific for SEB, CMV and different HIV peptide pools in five different SP subjects. (**D**) Shown is the homing molecule expression on matched CFSE^low^CD4^+^ T-cells specific for CMV *versus* HIV_NefGagPol_ peptide pool in four-five different SP subjects. Paired T-test p-values are indicated in the figures.

In a second experimental approach, antigen-specific CD4^+^ T-cells were identified based on their proliferation potential (CFSE^low^ phenotype) using the CFSE dilution assay (Figure 2A), as previously described [52]. The PBMCs from HIV-infected individuals were screened for the ability to respond to an antigen panel that included HIV Nef (n = 3), Gag (n = 6) and Pol (n = 11) overlapping peptide pools, HIV-p24 recombinant protein, SEB, and CMV-pp65 recombinant protein (Table 4). The PBMCs were then stimulated with the most immunogenic antigenic panel and the expression of integrin β7, CCR6, CXCR3, and CCR4 was quantified on CFSE^low^ CD4^+^ T-cells by polychromatic flow cytometry (Figure 2B). Similar to data obtained on HIV-specific CD154^+^CD4^+^ T-cells (Figure S1C), inter- and intra-donor variations were observed in the phenotype of CFSE^low^ CD4^+^ T-cells specific for different HIV peptide pools (Figure 2C). CXCR3 was expressed by the majority of antigen-specific cells, while the expression of integrin β7, CCR6 and CCR4 was limited to a fraction of cells (Figure 2C). The CD4^+^ T-cells proliferating in response to the HIV_NefGagPol_ peptide pool *versus* CMV from matched donors expressed significantly higher levels of integrin β7, CCR6, and CXCR3, with not significant differences regarding CCR4 expression (Figure 2D). The same trend was observed when CD4^+^ T-cells specific for all HIV peptide pools were compared with those specific for CMV from five different donors (Figure S2A). The HIV-specific CD4^+^ T-cells identified as CD154^+^ *versus* CFSE^low^ cells differed in the expression of homing receptors; these differences were observed when cells were stimulated with distinct (Nef *versus* Gag, respectively) or the same HIV antigenic pools (HIV-p24) (Figure S1C and Figure 2C). However, the expression at high levels of both integrin β7 and CCR6 remained a unique feature of HIV-specific CD4^+^ T-cells when compared to CMV-specific cells (Figures S1D and S2A, and Figure 2D). This unique particularity of HIV-specific CD4^+^ T-cells may confer them the ability to migrate into the GALT, a major site of HIV replication *in vivo* [8], [59].

CCR6 is a well established marker for Th17 and Th1Th17 cells with a CCR4^+^CCR6^+^ and CXCR3^+^CCR6^+^ phenotype [60]. Our results demonstrated that only a minority of CFSE^low^ HIV-specific CD4^+^ T-cells produced IL-17, while a larger fraction produced IFN-γ and TNF-α (Figure S3). These results indicate that HIV-specific CD4^+^ T-cells exhibit a Th1Th17 polarization profile. We previously demonstrated that a Th1Th17 profile is favorable to active HIV replication *in vitro* [44]. Therefore, HIV-specific cells from SP subjects may be highly permissive to HIV, as previously demonstrated in HIV progressors [41], [49], [54].

**Figure 3 pone-0032964-g003:**
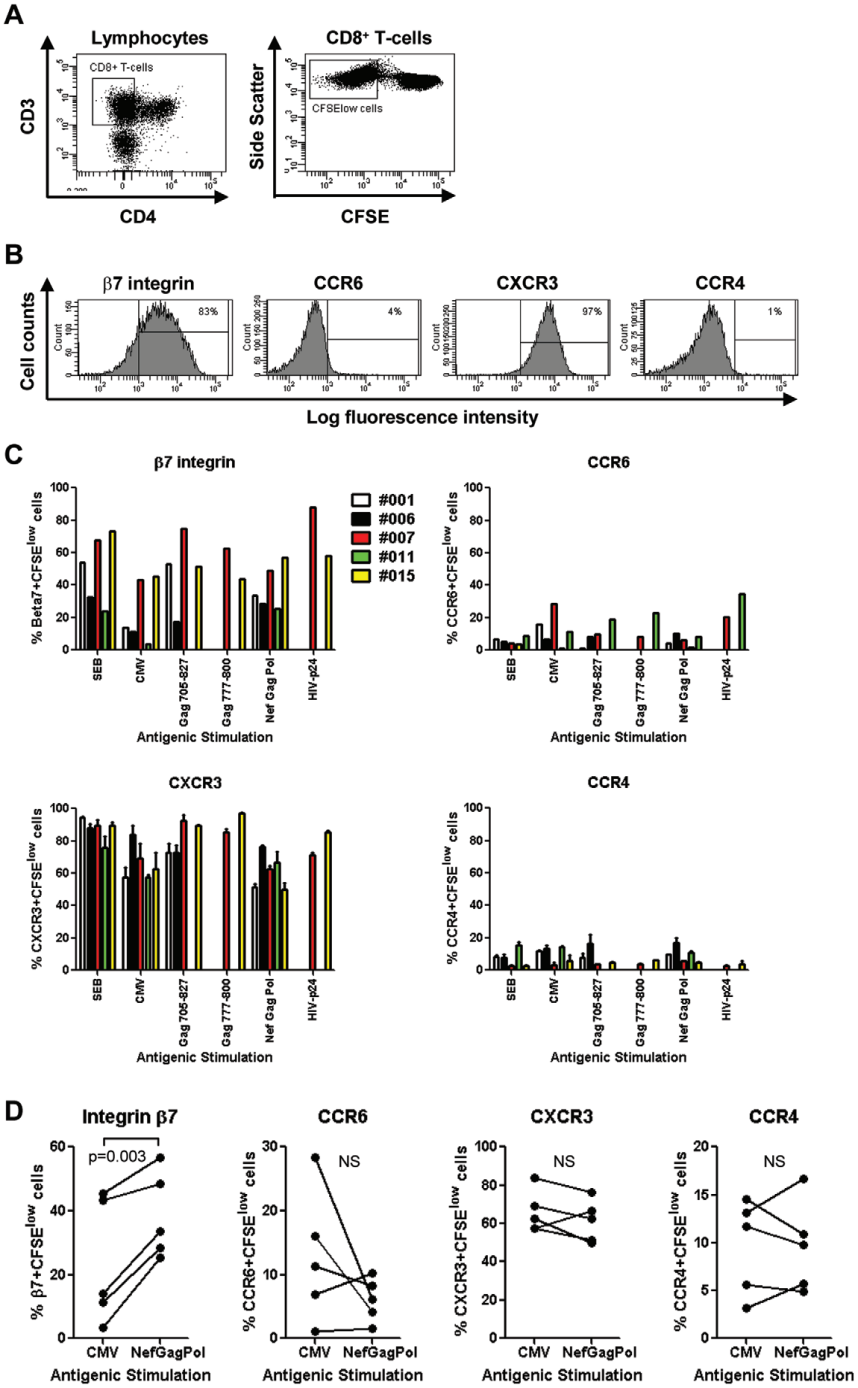
Homing potential of CD8^+^ T-cells proliferating in response to HIV peptides. PBMC from five SP subjects were stimulated and stained with Abs as in Figure 2. At day 6 of stimulation, cells were analyzed by polychromatic flow cytometry for (**A**) the frequency of CFSE^low^CD3^+^CD4^−^ T-cells (referred to as CD8^+^ T-cells) and (**B–D**) the expression of β7 integrin, CCR6, CXCR3, and CCR4 on CFSE^low^CD8^+^ T-cells. (**A–B**) Shown are results from one donor (i.e., SP 007) generated upon stimulation with HIV Gag_705–827_ peptide pool. (**C**) Shown is the expression of the homing receptors on CFSE^low^CD8^+^ T-cells specific for SEB, CMV, and different HIV peptide pools in five different SP subjects. (**D**) Shown is the homing molecule expression on matched CFSE^low^CD8^+^ T-cells specific for CMV *versus* HIV_NefGagPol_ peptide pool in five different SP subjects. Paired T-test p-values are indicated in the figures.

**The colocalization potential of HIV-specific CD4^+^ and CD8^+^ T-cells is mediated by the integrin β7 but not CCR6:** The antiviral properties of CD8^+^ T-cells are well characterized [19], [20], [21] and depend on their ability to colocalize in excess with target cells, such as CD4^+^ T-cells [28], [29]. We next investigated the expression of gut-homing molecules on HIV-specific CD8^+^ T-cells in order to evaluate their ability to colocalize with HIV-specific CD4^+^ T-cells for an efficient control of HIV replication *in vivo*. In preliminary experiments, we demonstrated that the majority of CD8^+^ T-cells exhibited a CD3^+^CD4^−^ phenotype (data not shown). Antigen-specific CD8^+^ T-cells were identified as CFSE^low^ cells (Figure 3A) and tested for their expression of the integrin β7, CCR6, CXCR3, and CCR4 (Figure 3B). Similar to antigen-specific CD4^+^ T-cells (Figure 2C), the expression of the homing receptors on CFSE^low^ CD8^+^ T-cells was subject to inter-donor variations (Figure 3C). Despite this heterogeneity, HIV_NefGagPol_-specific *versus* CMV-specific CFSE^low^ CD8^+^ T-cells from matched donors expressed significantly higher levels of integrin β7, while no significant differences were observed in the levels of CCR6, CXCR3, and CCR4 expression (Figure 3D). The same results were observed when CD8^+^ T-cells specific for all HIV peptide pools were compared with those specific for CMV (Figure S2B). These results suggest an increased ability of HIV-specific *versus* CMV-specific CD8^+^ T-cells to migrate *via* integrin β7 into the GALT, which is likely a site for the initial priming of HIV-specific T-cells.

**Figure 4 pone-0032964-g004:**
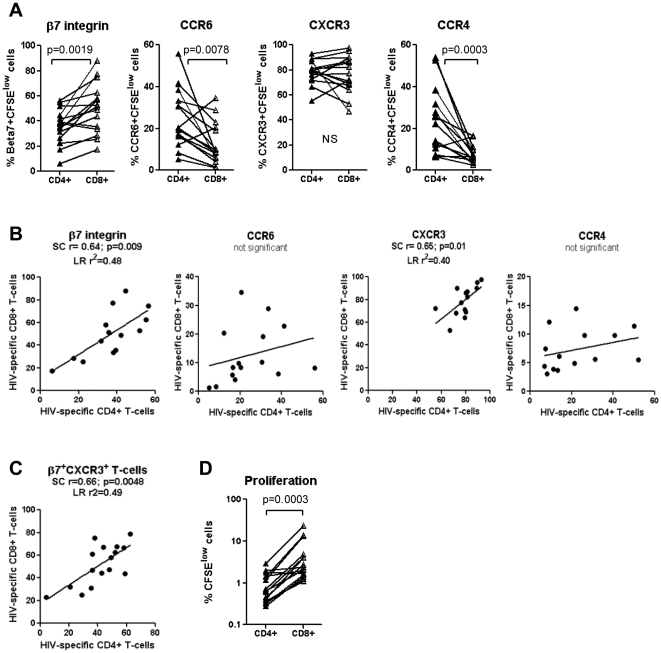
Potential colocalisation of HIV-specific CD8^+^ and CD4^+^ T-cells *via* integrin β7 but not CCR6. (**A–C**) PBMC from five SP subjects were stimulated with different HIV peptide pools or recombinant HIV-p24 and analyzed by polychromatic flow cytometry for the expression of homing molecules, as described in Figures 2 and 3. HIV-specific CD4^+^ and CD8^+^ T-cells were analyzed for (**A–C**) the expression of the homing molecules β7 integrin, CCR6, CXCR3, and CCR4 (**D**) the proliferation potential. (**A**) Shown is the expression of homing molecules on matched CD4^+^ *versus* CD8^+^ T-cells specific for different HIV peptide pools in five different SP subjects. Shown are correlations between (**B**) the frequency of matched HIV-specific CD4^+^ and CD8^+^ T-cells expressing β7 integrin, CCR6, CXCR3, or CCR4 alone, and (**C**) co-expressing the β7 integrin and CXCR3 molecules (β7^+^CXCR3^+^ phenotype). (**D**) Shown is the percentage of matched CD4^+^ *versus* CD8^+^ T-cells proliferating (CFSE^low^) in response to different HIV peptide pools. Paired T-test p-values are indicated in the Figures A and D. Spearman correlation r and p-values and linear regression r^2^ values are indicated in the Figures B and C.

To further investigate the colocalization potential of HIV-specific CD4^+^ and CD8^+^ T-cells, the frequency of cells expressing gut-homing molecules was compared between matched CD4^+^ and CD8^+^ T-cells proliferating in response to specific HIV peptide pools. Results depicted in Figure 4A show that HIV-specific CD8^+^ compared to CD4^+^ T-cells express significantly higher levels of integrin β7, lower levels of CCR6 and CCR4, and similarly high levels of CXCR3. Spearman correlation and linear regression models were applied and demonstrated a positive correlation between the frequency of HIV-specific CD4^+^ and CD8^+^ T-cells expressing the integrin β7 and CXCR3; this correlation was not observed for CCR6 and CCR4 (Figure 4B). Moreover, there was a positive correlation between the frequency of HIV-specific CD4^+^ and CD8^+^ T-cells co-expressing the integrin β7 and CXCR3 (Figure 4C). Furthermore, the β7^+^CXCR3^+^ phenotype appears to be a unique feature of HIV-specific T-cells since the frequency of β7^+^CXCR3^+^ cells was significantly higher in HIV_NefGagPol_-specific *versus* CMV-specific CD4^+^ and CD8^+^ T-cells from four matched SP subjects (Figure S2C). The proliferation of CD8^+^ compared to CD4^+^ T-cells in response to a specific HIV peptide pool was significantly higher in all five HIV-infected subjects (Figure 4D), with median CD8/CD4 ratios of 3.2, 2.3, 4.1, 5.6, and 2.8 in SP subjects 001, 006, 007, 011, and 015, respectively (data not shown). These results suggest that a significant fraction of HIV-specific CD8^+^ T-cells may colocalize with CD4^+^ T-cells (at high CD8/CD4 ratios) in certain anatomic sites of the GALT where homing depends on integrin β7 and CXCR3. In contrast, HIV-specific CD8^+^ T-cells express low levels of CCR6 and CCR4 and thus may be impaired in their ability to colocalize and control viral replication in CCR6^+^CCR4^+^ CD4^+^ T-cells, such as Th17 cells [51] which are highly permissive to infection [44], [45]. Thus, the low expression of CCR6 on CD8^+^ T-cells may reflect their limited ability to control HIV replication in CD4^+^ T-cells from certain GALT sites such as the Peyer's Patches, where homing depends on CCR6 [32], [33], [34].

**Figure 5 pone-0032964-g005:**
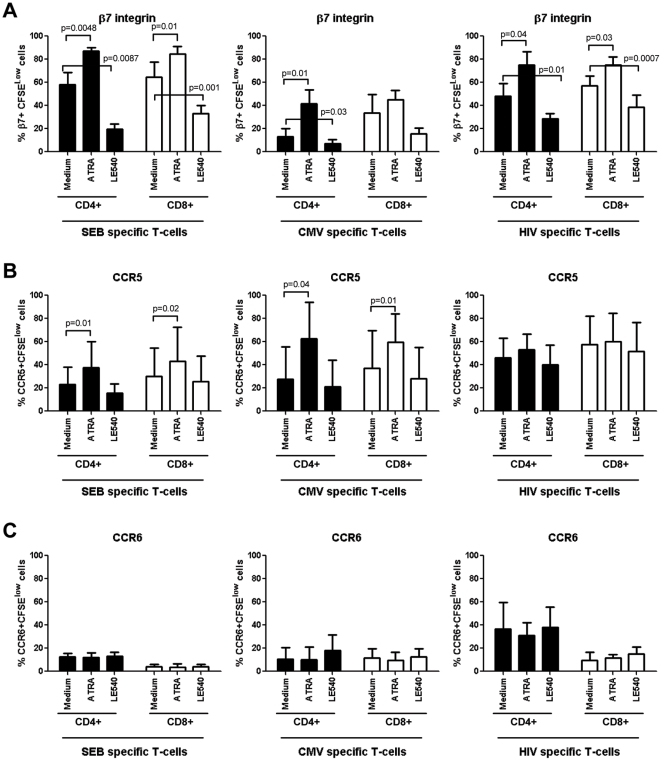
Retinoic acid activation pathway regulates integrin β7 expression on HIV-specific T-cells. PBMC from SP subjects were loaded in CFSE (0.5 µM) and stimulated with HIV_Nef-Gag-Pol_ peptide pool (500 ng/ml), SEB (25 ng/ml), or the recombinant CMV-pp65 peptide pool (1 µg/ml) for 6 days at 37°C in the presence or absence of all-trans-retinoic acid (ATRA; 10 nM) or the RA antagonist LE540 (1 µg/ml). Cells were stained with a cocktail of fluorochrome-conjugated CD3, CD4, integrin β7, CCR5 or CCR6 Abs and analyzed for the expression of (**A**) integrin β7, (**B**) CCR5, and (**C**) CCR6 on CFSE^low^ CD4^+^ and CD8^+^ T-cells specific for SEB, CMV, and HIV antigens. Experiments were performed with cells from four SP subjects (mean±SD). Paired t-Test p-values are indicated in the figures.

**The retinoic acid pathway regulates expression of integrin β7 but not CCR6 and CCR5 on HIV-specific T-cells:** The imprinting for gut-homing is regulated at least in part by RA, a derivate of vitamin A metabolism produced by the intestinal dendritic cells [46], [47]. Of note, exposure of CD4^+^ T-cells to RA upregulates integrin β7 and CCR5 expression and renders them highly permissive to HIV replication [61], [62]. Here, we investigated whether RA can be used to manipulate the colocalization potential of HIV-specific CD4^+^ and CD8^+^ T-cells *via* integrin β7 and CCR6. With this in mind, PBMC from SP subjects were exposed to the HIV_NefGagPol_ peptide pool, SEB, or CMV in the presence or absence of all-trans RA (ATRA) or the RA antagonist LE540. In preliminary experiments, we demonstrated that at physiological dose (10 nM [61], [63]), ATRA did not have any significant effect on cell proliferation, while LE540 at 1 µg/ml [64] decreased integrin β7 expression on SEB-specific T-cells without interfering with cell viability (data not shown). The expression of integrin β7, CCR5, and CCR6 was quantified on CFSE^low^ T-cells by multicolor flow cytometry. Exposure to ATRA and LE540 led to a significant increase and decrease, respectively, in the integrin β7 expression on CD4^+^ T-cells specific for HIV-, SEB, and CMV and also on CD8^+^ T-cells specific for HIV and SEB (Figure 5A). The ATRA also increased expression of CCR5 on SEB-specific and CMV-specific but not on HIV-specific T-cells (Figure 5B), where levels of CCR5 expression were higher, although not statistically significant due to donor-to-donor variability, compared to those on SEB-specific and CMV-specific T-cells (Paired t-Test p = 0.04 and p = 0.1, respectively). In contrast, ATRA and LE540 treatment did not interfere with the expression of CCR6 on antigen-specific CD4^+^ or CD8^+^ T-cells (Figure 5C). These results demonstrate that the RA pathway regulates the expression of integrin β7 but does not interfere with CCR5 and CCR6 expression on HIV-specific CD4^+^ and CD8^+^ T-cells.

**Figure 6:**
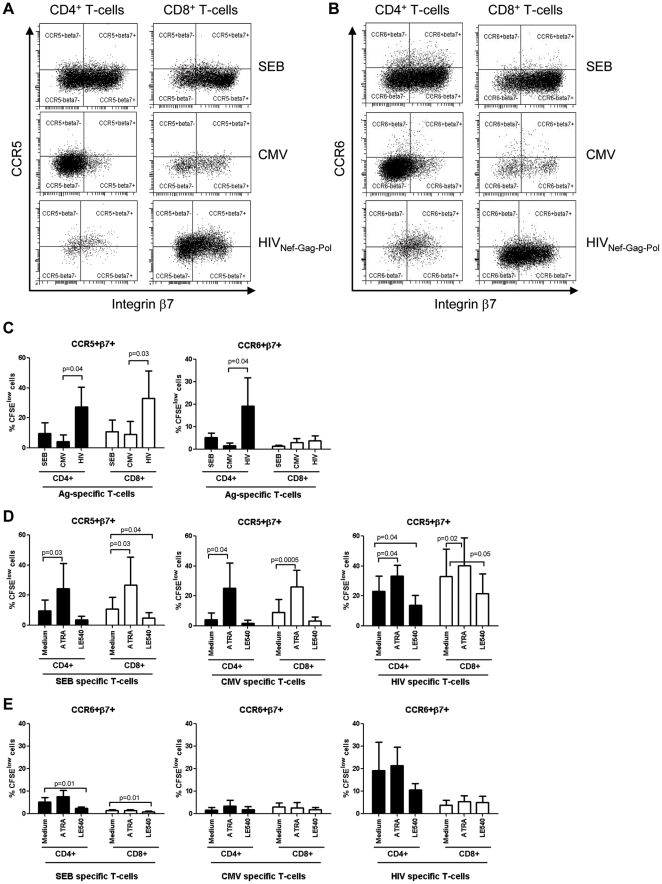
Retinoic acid upregulates the frequency of HIV-specific T-cells with a β7^+^CCR5^+^ but not β7^+^CCR6^+^ phenotype. PBMC from SP subjects were loaded in CFSE (0.5 µM) and stimulated as in Figure 5. Cells were stained with a cocktail of fluorochrome-conjugated CD3, CD4, and integrin β7, and CCR5 or CCR6 Abs. CFSE^low^ CD4^+^ and CD8^+^ T-cells specific for SEB, CMV, and HIV_Nef-Gag-Pol_ peptide pool were analyzed for the co-expression of (**A**) integrin β7 and CCR5 and (**B**) integrin β7 and CCR6. The effects of RA and LE540 on the frequency of Ag-specific CD4^+^ and CD8^+^ T-cells exhibiting a β7^+^CCR5^+^ or β7^+^CCR6^+^ phenotype were then analyzed. (**A–B**) Shown are results from one representative SP subject and (**C–E**) statistical analysis of results from experiments performed with cells from five SP subjects (mean±SD). Paired t-Test p-values are indicated in the figures.

To gain more insights into the regulation of trafficking potential of HIV-specific T-cells, we studied the effects of RA and LE540 on the frequency of HIV-specific T-cells with a β7^+^CCR5^+^ and β7^+^CCR6^+^ phenotype. The CD4^+^ and CD8^+^ T-cells proliferating in response to HIV differed from CMV- or SEB-specific T-cells by an increased frequency of β7^+^CCR5^+^ T-cells (Figure 6A and C). The pool of HIV-specific CD4^+^ T-cells included higher frequencies of cells with a β7^+^CCR6^+^ phenotype compared to cells specific for CMV or SEB, while CD8^+^ T-cells specific for HIV, CMV, or SEB included very low frequencies of β7^+^CCR6^+^ T-cells (Figure 6B). Exposure to ATRA and LE540 led to a significant increase and decrease, respectively, in the frequency of HIV-specific CD4^+^ and CD8^+^ T-cells with a β7^+^CCR5^+^ phenotype (Figure 6D). Since ATRA does not modulate CCR5 expression on HIV-specific cells (Figure 5C), the increase in the frequency of β7^+^CCR5^+^ cells (Figure 6D) is likely due to the upregulation of integrin β7 expression on existing CCR5^+^ T-cells. Finally, ATRA and LE540 treatment had no significant effects on the frequencies of HIV-specific CD4^+^ or CD8^+^ T-cells with a β7^+^CCR6^+^ phenotype (Figure 6E). These results provide evidence that ATRA may be used to enhance recruitment of HIV-specific CD4^+^ and CD8^+^ T-cells across the intestinal endothelium *via* the integrin β7. In contrast, ATRA does not interfere with CCR6 expression and cannot facilitate the *in situ* colocalization of CD8^+^ T-cells with CCR6^+^CD4^+^ T-cells.

## Discussion

**Figure 7:**
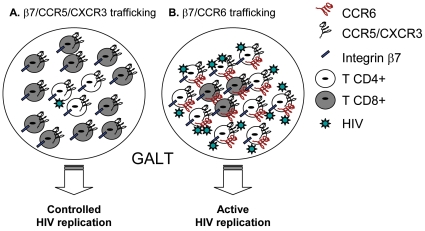
Proposed model for the differential colocalization of HIV-specific CD4^+^ and CD8^+^ T-cells into the GALT. The control of viral replication is dependent on the *in situ* colocalization of excess effector *versus* target cells [28]. Given the results included in Figures 1–6 of the present manuscript, we propose a model where (**A**) HIV replication in CD4^+^ T-cells may be controlled by CD8^+^ T-cells in certain GALT sites (*e.g.*, lamina propria), where recruitment is dependent on integrin β7, CXCR3 and CCR5 because of an increased ratio between HIV-specific CD8^+^ and CD4^+^ T-cells. In contrast, (**B**) HIV-specific CD4^+^ T-cells recruited into other GALT sites *via* CCR6 (*e.g.*, Peyers's Patches) may escape the CD8^+^ T-cell-mediated antiviral control due to a limited CCR6-dependent colocalization potential of CD4^+^ and CD8^+^ T-cells. This model is in line with our previous findings that CCR6^+^CD4^+^ T-cells harbor the highest levels of integrated HIV-DNA *in vivo* [44] and suggests that novel therapeutic strategies aimed at increasing CCR6 expression on CD8^+^ T-cells may lead to a better control of HIV replication in CCR6^+^CD4^+^ T-cells.

The GALT is a major site for HIV replication *in vivo* [8], [59], with HIV-specific CD4^+^ T-cells being highly permissive to infection [49]. The recruitment of effector CD8^+^ T-cells in the proximity of target CD4^+^ T-cells is a prerequisite for an efficient control of viral replication *in viv*o [29], [65]. In this study, we investigated the potential of HIV-specific CD8^+^ T-cells to colocalize in excess with CD4^+^ T-cells in the GALT and explored the role of the retinoic acid (RA) activation pathway in regulating this process. We demonstrated that a large fraction of HIV-specific CD4^+^ and CD8^+^ T-cells express the gut-homing molecules integrin β7 and CXCR3 while CCR6, a marker of HIV permissiveness in CD4^+^ T-cells [44], [48], was expressed at high and low levels on HIV-specific CD4^+^ and CD8^+^ T-cells, respectively. We also demonstrated that RA upregulated integrin β7 expression but did not affect CCR6 expression. Our data support a model in which HIV-specific CCR6^+^CD4^+^ T-cells escape the antiviral control of CD8^+^ T-cells in certain GALT sites (*e.g.*, Peyer's Patches) where migration is dependent on CCR6 [32], [33], [34] (Figure 7). Considering the critical role played by CCR6^+^ Th17 cells in mucosal immunity [66], uncontrolled HIV replication in these cells likely leads to dramatic alterations of mucosal immunity and microbial translocation [13], [59], [67], [68], [69]. These observations were made in a cohort of SP subjects with a median time since infection 16 years. Whether CD8^+^ T-cells colocalize in excess with CCR6^+^CD4^+^ T-cells for an efficient control of HIV replication in SP subjects during the first years of infection or in HIV-exposed uninfected individuals remains to be determined.

Our initial hypothesis was that the colocalization potential of CD8^+^ T-cells with CCR6^+^CD4^+^ T-cells was altered in HIV-infected subjects with disease progression but not in slow progressors (SP). To test this hypothesis, the expression of CCR6 was quantified on peripheral blood CD8^+^ and CD4^+^ T-cells from SP subjects and two other cohorts of HIV progressors, recently infected untreated (RI) and chronically infected under viral suppressive ART subjects (CI on ART). Unexpectedly, we found an alteration in the frequency of CCR6-expressing CD8^+^ and CD4^+^ T-cells in HIV-infected subjects regardless of their clinical characteristics of disease progression. This suggested the inability of CD8^+^ T-cells to be recruited in excess in the proximity of CCR6^+^CD4^+^ T-cells, and this even in subjects with slow disease progression. This is consistent with studies reporting immunological alterations related to HIV persistence in CD4^+^ T-cells and chronic immune activation in SP subjects, especially after many years of infection in the absence of ART [23], [24], [70], [71].

According to the paradigm of tissues-specific homing, effector memory T-cells are imprinted with the ability to recirculate into peripheral tissues where the initial antigen encounter took place [37], [46], [56], [72]. A specific combination of adhesion molecules and chemokine receptors regulate the multi-step process of tissue-specific homing of T-cells [73], [74]. Also, local presentation of antigens by endothelial cells contribute to the recruitment of T-cells into specific sites [75]. In this study, we demonstrated that HIV-specific CD154^+^CD4^+^ T-cells express molecules regulating migration into the GALT (integrin β7, CCR6, CXCR3) and skin (CCR4). Considering the fact that the GALT [59], [69] but not the skin are sites of HIV replication *in vivo*, this broad homing potential of HIV-specific CD154^+^CD4^+^ T-cells was unexpected and inconsistent with the paradigm of tissue-specific homing of pathogen-specific T-cells. This exception from the rule is not unique. In fact, T-cells induced upon subcutaneous yellow fever immunization have a dynamic migration program and home into multiple distal tissues, including the GALT [76].

CD154 was proposed as a surrogate marker for the identification of cytokine-producing T-cells in response to an antigenic stimulation [58]. However, we found a differential expression of homing molecules on recently activated CD154^+^ compared to proliferating CD4^+^ T-cells (CFSE^low^) in response to HIV, SEB, or CMV. The skin-homing receptor CCR4 was highly expressed on CD154^+^ compared to CFSE^low^ CD4^+^ T-cells specific for HIV and CMV. This is consistent with the paradigm that T-cells are originally imprinted with a skin-homing potential that is lost during the process of differentiation into specialized Ag-specific cells [46]. Also, the expression of CCR6 was higher on CMV-specific CD154^+^ compared to CFSE^low^ CD4^+^ T-cells, while the integrin β7 expression was lower on SEB-specific CD154^+^ compared to CFSE^low^ CD4^+^ T-cells. The finding that some antigens induced either CD154^+^ or CFSE^low^ CD4^+^ T-cells but not both suggests that CD154 expression does not predict the ability of a cell to proliferate. Accordingly, CD154^+^CD4^+^ T-cells were mainly triggered by HIV_Nef_ peptide pools, while CFSE^low^CD4^+^ T-cells were selectively induced by HIV_Gag_ peptide pools (Table 4 and Table S1). Thus, HIV-specific CD154^+^ and CFSE^low^ CD4^+^ T-cells exhibit distinct homing potential and antigenic specificity and therefore may represent different stages of CD4^+^ T-cell differentiation with distinct roles in antiviral immunity. The molecular determinism underlying these differences remains unclear but might be related to the anatomic site of original antigenic priming.

The HIV establishes a persistent infection by mechanisms that are not clearly understood, and viral eradication is not achieved under current antiretroviral therapies [14], [77]. The CD4^+^ T-cells play a critical role in HIV pathogenesis [77]. The HIV-specific compared to CMV-specific CD4^+^ T-cells are preferentially infected with HIV *in vivo* [49]. This is because HIV-specific CD4^+^ T-cells express high levels of the HIV coreceptor CCR5 [41] and produce low levels of CCR5 binding chemokines and therefore fail to protect themselves from infection in an autocrine manner [54]. Consistent with the evidence that the GALT is a major site of HIV replication [59], [69], we observed that HIV-specific compared to CMV-specific CD154^+^CD4^+^ T-cells expressed at high levels both gut-homing molecules integrin β7 and CCR6. We also observed increased expression of integrin β7 and CCR6 on CD4^+^ T-cells proliferating in response to HIV compared to CMV antigens. In addition to their role in gut-homing, the integrin β7 was identified as a new HIV-gp120 binding receptor [61], [62], and its expression on HIV-specific CD4^+^ T-cells might favor HIV binding on these cells and viral dissemination from the portal sites of entry. The CCR6 is a marker for memory CD4^+^ T-cells with a Th17 and Th1Th17 lineage polarization profile [51]. We found that a small fraction of HIV-specific cells produced IL-17, with the majority of cells producing IFN-γ and TNF-α. Thus, HIV-specific CD4^+^ T-cells exhibit a Th1Th17 polarization profile. Indeed other studies demonstrated that very few HIV-specific CD4^+^ T-cells produce IL-17 [78]. Considering our previous findings that HIV replicates actively in T-cells with a Th1Th17 polarization profile [44], these results suggest that HIV-specific CD4^+^ T-cells in SP subjects are also permissive to infection. Consistent with this prediction, the expression of the HIV co-receptor CCR5 was relatively high on HIV-specific CD4^+^ T-cells from SP subjects. This may render them extremely permissive to infection and may explain why some of the SP subjects begin loosing their CD4 counts, especially after many years of infection in the absence of ART [16]. Hence, the expression of integrin β7, CCR6, and CCR5 represents a unique “*signature*” for HIV-specific T-cells. The relationship between imprinting for gut-homing and viral permissiveness was recently demonstrated for adenovirus serotype 5 (AD5)-specific CD4^+^ T-cells generated upon AD5-HIV vaccination (STEP trial), as cells exhibited an integrin α4β7^+^CCR5^+^ phenotype and high susceptibility to HIV infection [79]. The molecular mechanisms that control homing potential of HIV-specific T-cells are likely related to the cellular/tissue environment in which these cells initially encountered antigen. Indeed, the GALT dendritic cells produce RA, which is known to trigger integrin α4β7 expression and upregulate CCR5 expression on T-cells [47], [48], [61]. Similarly, the GALT environment is rich in Th17 polarizing cytokines (TGF-β, IL-1, IL-6) [51], [80], [81] that may trigger CCR6 expression on HIV-specific CD4^+^ T-cells.

The CD8^+^ T-cells control HIV replication in target cells *via* cytotoxic and non-cytotoxic mechanisms [19], [20], [21]. Recent studies using visualization techniques demonstrated that recruitment of excess viral-specific effectors in the vicinity of target cells is critical for the control of viral replication and disease progression in an SIV model of infection [29]. Our results reveal that matched HIV-specific CD8^+^ and CD4^+^ T-cells may colocalize to anatomic sites where recruitment is mediated by integrin β7, CXCR3 and/or CCR5 (Figure 7A). The expression of integrin β7 on both HIV-specific CD8^+^ and CD4^+^ T-cells supports the idea that these cells are primed with the antigen within the GALT, where they are likely exposed to factors that imprint cells with a gut-homing potential [46], [47]. The CXCR3 is responsible for leukocyte migration into the inflammatory sites, including the gut [56]. Of note, a decreased frequency of CXCR3^+^CD8^+^ T-cells was reported in advanced HIV-1 infection that might contribute to cytotoxic T-lymphocyte dysfunction [82]. In our SP cohort, HIV-specific T-cells expressed maximal levels of CXCR3, and this suggests their functional competence *in vivo*. In contrast, we observed that CCR6 was expressed at high levels on HIV-specific CD4^+^ but not CD8^+^ T-cells. The low expression of CCR6 on HIV-specific CD8^+^ T-cells was consistent with the decreased frequency of memory CCR6^+^CD8^+^ T-cells in the peripheral blood of HIV-infected subjects with slow and rapid disease progression compared to uninfected individuals. This points to the fact that regardless of the clinical characteristics of HIV disease progression, CD8^+^ T-cells have a limited ability to colocalize with CCR6^+^CD4^+^ T-cells and therefore to control HIV replication in these cells. This may explain in part why CCR6^+^CD4^+^ T-cells are highly permissive to HIV-DNA integration *in vivo* [44]. The escape of CCR6^+^CD4^+^ T-cells from the non-cytotoxic antiviral control by CD8^+^ T-cells may also explain the preferential depletion of these cells during disease progression [44], likely *via* a virus-induced toxicity mechanism [83].

Several previous studies demonstrated that HIV-specific CD8^+^ T cells from SP subjects are efficient in controlling viral replication *ex vivo* [15], [16], [19]. This is consistent with our observations *in vitro* that HIV replication in antigen-stimulated PBMC from the seven SP subjects was controlled (undetectable HIV-p24 levels, as quantified by ELISA), likely by CD8^+^ T-cells (Ancuta, unpublished observations). However, the situation *in vivo* may be different. Our results support a model in which the low expression of CCR6 on HIV-specific CD8^+^ T-cells is exploited by HIV-1 for its efficient replication in CCR6^+^CD4^+^ T-cells in some GALT sites such as the Peyer's Patches (Figure 7B). In this context, it is of interest to identify ways to increase the ability of HIV-specific CD8^+^ T-cell to colocalize with HIV-specific CD4^+^ T-cells. We found that RA increased the expression of integrin β7 on T-cells specific for HIV, CMV and SEB and the frequency of T-cells specific for CMV and SEB with a β7^+^CCR5^+^ phenotype. The HIV-specific CD4^+^ and CD8^+^ T-cells included a relatively high fractions of β7^+^CCR5^+^, and their frequency remained high upon RA treatment. In contrast, HIV-specific CD4^+^ compared to CD8^+^ T-cells included an increased frequency of cells with a β7^+^CCR6^+^ phenotype and the physiological dose of RA used (10 nM, [61], [63]) did not upregulate CCR6 expression on CD8^+^ nor CD4^+^ T-cells. Other factors responsible for Th17 differentiation, such as TGF-β, IL-1, IL-6 [51], [80], [81], might be involved in regulating CCR6 expression on HIV-specific CD8^+^ T-cells and remain to be identified.

Together, these results suggest the ability of HIV-specific CD8^+^ and CD4^+^ T-cells to colocalize into the GALT (*e.g.*, lamina propria) *via* integrin β7, CCR5, and CXCR3 and reveal a limited potential of HIV-specific CD8^+^ T-cells to migrate into other GALT sites (*e.g.*, Peyer's Patches), where recruitment is dependent on CCR6 [32], [33], [34]. Studies on gut biopsies are required to validate our proposed model (Figure 7), which is consistent with the preferential permissiveness of CCR6^+^CD4^+^ T-cells to HIV infection *in vivo* [44]. In this context, understanding molecular mechanisms regulating CCR6 expression on HIV-specific CD8^+^ T-cells, together with the expression of the CCR6 ligands into the GALT, is of paramount importance for the design of new therapeutic strategies aimed at HIV eradication. However, caution must be taken when designing such strategies to avoid an increased recruitment of HIV targets at the portal sites of mucosal entry. Finally, studies evaluating the functionality of the immune system in response to ART and vaccination may gain in physiological relevance if they consider monitoring the *in situ* colocalization potential of HIV-specific CD8^+^ and CD4^+^ T-cells as a new correlate of protection.

## Supporting Information

Figure S1 **The HIV-specific** ***versus*** **CMV-specific CD154^+^CD4^+^ T-cells preferentially express a gut-homing potential.** PBMC from SP subjects were stimulated with different HIV Nef, Gag, Pol peptide pools (10 µg/ml), recombinant HIV-p24 protein (5 µg/ml), SEB (1 µg/ml), or CMV-pp65 peptide pool (5 µg/ml) for 18 hours at 37°C in the presence of fluorescence conjugated anti-CD154-PE/Cy5 Abs (20 µl/2×10^6^ cells/0.2 ml/well). Antigen-specific T-cells were identified as CD154^+^ cells, as previously described [58]. Cells were harvested, stained with a cocktail of fluorochrome-conjugated CD3, CD4, and β7 integrin, CCR6, CXCR3, or CCR4 Abs and analyzed by polychromatic flow cytometry for **(A)** the expression of CD154 on CD3^+^CD4^+^ T-cells and **(B–D)** the expression of homing molecules on CD3^+^CD4^+^CD154^+^ T-cells. **(A–B)** Shown are results from one SP subjects (SP 015 stimulated with the HIV Nef_5164-5187_ peptide pool), representative of results generated with cells from five different donors. **(C)** The expression (%) of homing receptors was analyzed on CD154^+^ T-cells specific for SEB, CMV, and different HIV peptide pools in five different SP subjects. **(D)** Shown are statistical analyses of the homing molecule expression on CD154^+^CD4^+^ T-cells specific for SEB, CMV, and HIV (all peptide pools) in five different SP subjects (box & whisker graph: range and median). Mann-Whitney p-values are indicated in the figures.
(TIF)

Figure S2 **Homing potential of CD4^+^ and CD8^+^ T-cells proliferating in response to HIV peptides.** PBMC from SP subjects were stimulated with different antigens and analyzed by polychromatic flow cytometry for the expression of homing molecules as in Figures 2 and 3. Shown are statistical analyses of the homing molecule expression on **(A)** CFSE^low^CD4^+^ and **(B)** CFSE^low^CD8^+^ T-cells specific for SEB, CMV, and HIV (all peptide pools) in five different SP subjects (box & whisker graph: range and median). Mann-Whitney p-values are indicated in the figures. **(C)** Shown are statistical analyses of the integrin β7 and CXCR3 co-expression on matched CD4^+^ and CD8^+^ T-cells proliferating (CFSE^low^) in response to CMV *versus* HIV_NefGagPol_ peptide pool in four different SP subjects. Paired T-test p-values are indicated in the figures.
(TIF)

Figure S3 **The HIV-specific CD4^+^ T-cells exhibit a Th1Th17 polarization profile.** PBMCs from SP subjects were loaded in CFSE (0.5 µM) and stimulated with different HIV Nef, Gag, Pol peptide pools (500 ng/ml), recombinant HIV-p24 (5 µg/ml), SEB (25 ng/ml), a peptide pool spanning the CMV pp65 protein (1 µg/ml), or *C. albicans hyphae* (25 µl of protein lysate) for 5 days at 37°C and further stimulated with PMA (50 ng/ml) and Ionomycin (1 µg/ml) in the presence of Brefeldin A (10 µg/ml) for 18 hours at 37°C. Cells were stained on the surface with CD3 and CD8 Abs as well as intracellularly with IFN-γ, TNF-α, and IL-17 Abs and then analyzed by polychromatic flow cytometry for the expression of cytokines in CD3^+^CD8^−^ (referred as CD4^+^ T-cells) cells. Shown is **(A)** the gating strategy for CD4^+^ T-cells identification and **(B)** representative dot plots of IFN-γ, TNF-α, and IL-17 production by HIV-specific and *C. albicans*-specific CD4^+^ T-cells. **(C)** Shown is the intracellular expression of cytokines by CFSE^low^CD4^+^ T-cells specific for SEB, CMV, *C. albicans*, and different HIV peptide pools in five different SP subjects.
(TIF)

Table S1 **Screening for HIV-1 specific CD4^+^ T-cells responses using the CD154 co-culture assay.** To identify antigen-specific CD4^+^ T-cells, the CD154/CD40L assay was performed as previously described (89). To this aim, PBMC from five HIV-infected SP subjects were stimulated with SEB (1 µg/ml), CMV-pp65 peptide pool (5 µg/ml), recombinant HIV-p24 protein (5 µg/ml), or HIV peptide pools (10 µg/ml) in the presence of anti-CD154-PE/Cy5 Abs (20 µl/well) and monensin (2 µM) for 16 hrs at 37°C. Cells were then harvested, stained for surface markers with fluorescence-conjugated Abs against CD3, CD4, integrin β7, CCR6, CXCR3, and CCR4, and analyzed by flow cytometry for the expression of homing markers on CD3^+^CD4^+^CD154^+^ T-cells. Results are expressed as % of CD4^+^CD154^+^ T-cells. Values included in the table were >1.5-fold higher than the background CD154 expression observed for cells cultured in the absence of antigen.
(DOC)
