# Supplementary figures and images for: The Colocalization Potential of HIV-Specific CD8+ and CD4+ T-Cells is Mediated by Integrin β7 but Not CCR6 and Regulated by Retinoic Acid
Source: PLoS One. 2012 Mar 28;7(3):e32964. doi: 10.1371/journal.pone.0032964 (PMC3314661; doi:10.1371/journal.pone.0032964)

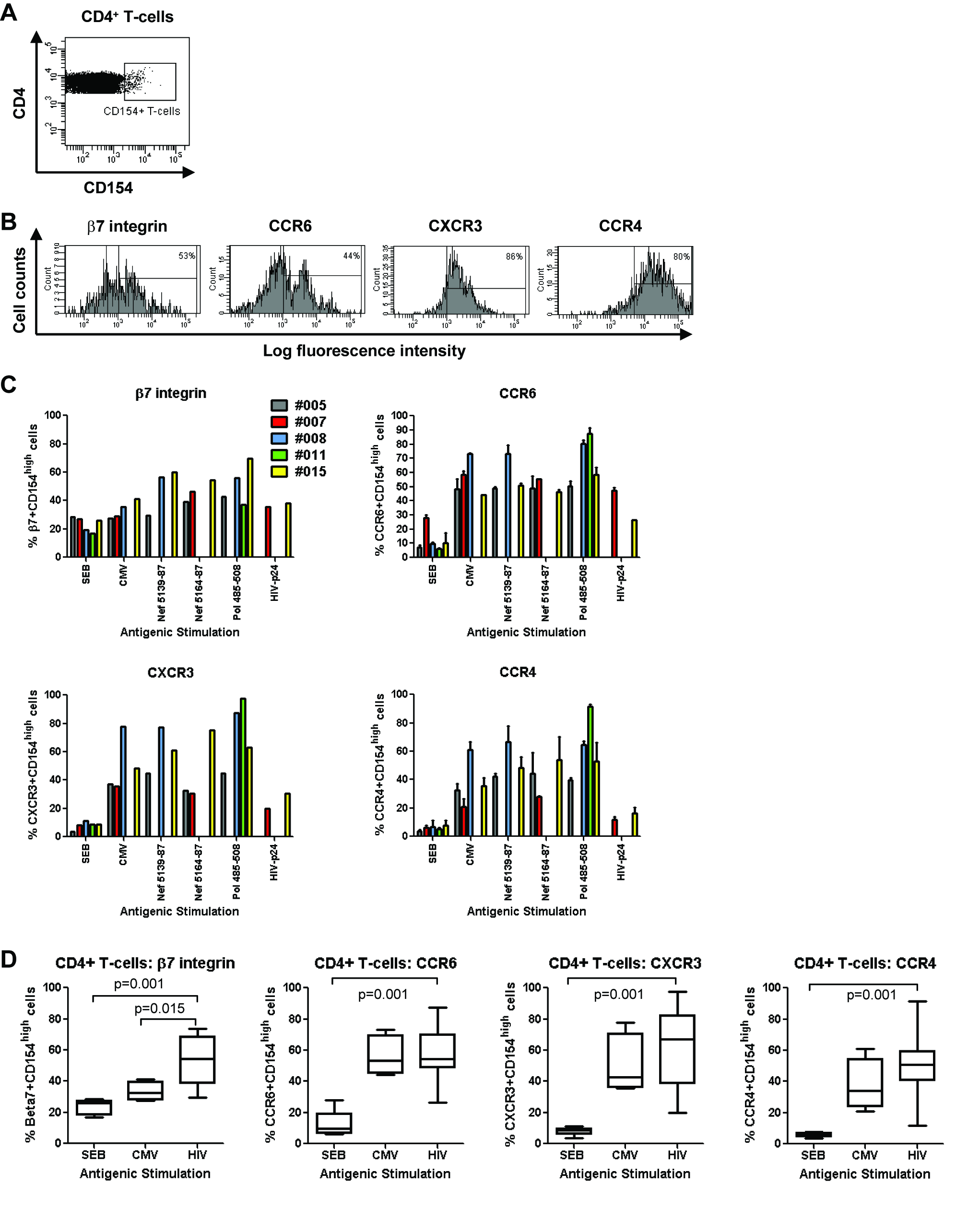

Supplement: Figure S1 — The HIV-specific versus CMV-specific CD154+CD4+ T-cells preferentially express a gut-homing potential. PBMC from SP subjects were stimulated with different HIV Nef, Gag, Pol peptide pools (10 µg/ml), recombinant HIV-p24 protein (5 µg/ml), SEB (1 µg/ml), or CMV-pp65 peptide pool (5 µg/ml) for 18 hours at 37°C in the presence of fluorescence conjugated anti-CD154-PE/Cy5 Abs (20 µl/2×106 cells/0.2 ml/well). Antigen-specific T-cells were identified as CD154+ cells, as previously described [58]. Cells were harvested, stained with a cocktail of fluorochrome-conjugated CD3, CD4, and β7 integrin, CCR6, CXCR3, or CCR4 Abs and analyzed by polychromatic flow cytometry for (A) the expression of CD154 on CD3+CD4+ T-cells and (B–D) the expression of homing molecules on CD3+CD4+CD154+ T-cells. (A–B) Shown are results from one SP subjects (SP 015 stimulated with the HIV Nef5164-5187 peptide pool), representative of results generated with cells from five different donors. (C) The expression (%) of homing receptors was analyzed on CD154+ T-cells specific for SEB, CMV, and different HIV peptide pools in five different SP subjects. (D) Shown are statistical analyses of the homing molecule expression on CD154+CD4+ T-cells specific for SEB, CMV, and HIV (all peptide pools) in five different SP subjects (box & whisker graph: range and median). Mann-Whitney p-values are indicated in the figures. (TIF) [file pone.0032964.s001.tif]

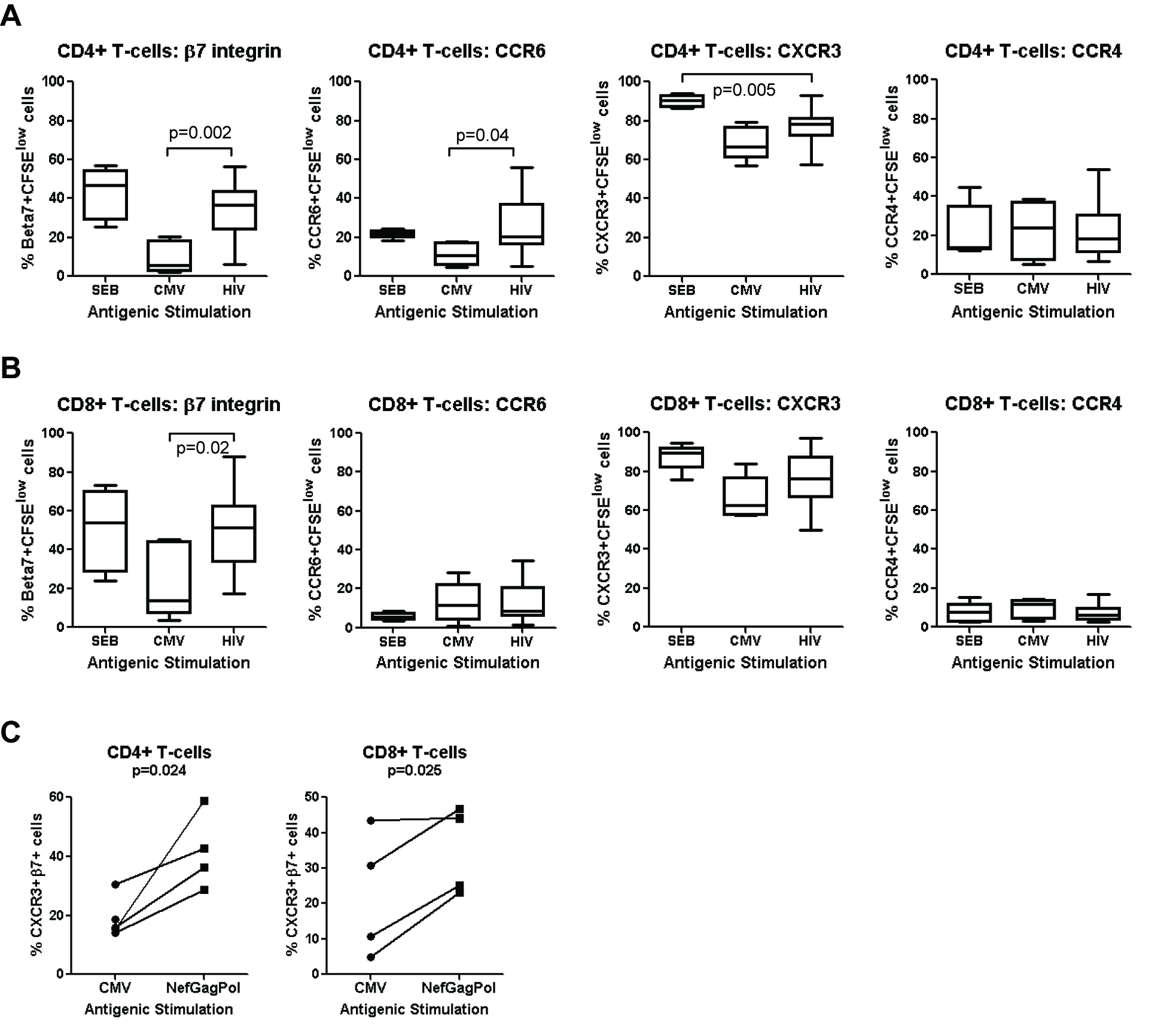

Supplement: Figure S2 — Homing potential of CD4+ and CD8+ T-cells proliferating in response to HIV peptides. PBMC from SP subjects were stimulated with different antigens and analyzed by polychromatic flow cytometry for the expression of homing molecules as in Figures 2 and 3. Shown are statistical analyses of the homing molecule expression on (A) CFSElowCD4+ and (B) CFSElowCD8+ T-cells specific for SEB, CMV, and HIV (all peptide pools) in five different SP subjects (box & whisker graph: range and median). Mann-Whitney p-values are indicated in the figures. (C) Shown are statistical analyses of the integrin β7 and CXCR3 co-expression on matched CD4+ and CD8+ T-cells proliferating (CFSElow) in response to CMV versus HIVNefGagPol peptide pool in four different SP subjects. Paired T-test p-values are indicated in the figures. (TIF) [file pone.0032964.s002.tif]

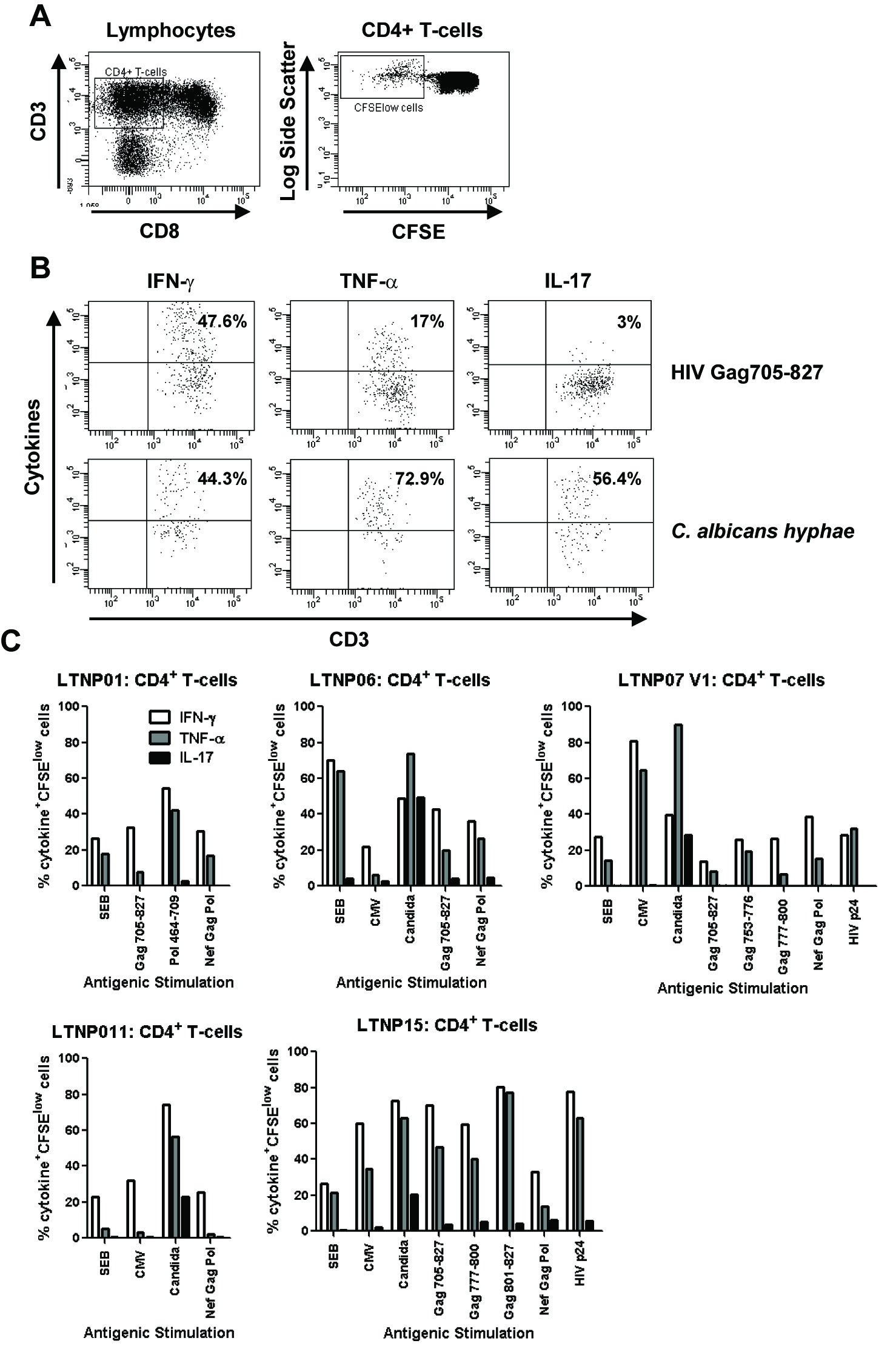

Supplement: Figure S3 — The HIV-specific CD4+ T-cells exhibit a Th1Th17 polarization profile. PBMCs from SP subjects were loaded in CFSE (0.5 µM) and stimulated with different HIV Nef, Gag, Pol peptide pools (500 ng/ml), recombinant HIV-p24 (5 µg/ml), SEB (25 ng/ml), a peptide pool spanning the CMV pp65 protein (1 µg/ml), or C. albicans hyphae (25 µl of protein lysate) for 5 days at 37°C and further stimulated with PMA (50 ng/ml) and Ionomycin (1 µg/ml) in the presence of Brefeldin A (10 µg/ml) for 18 hours at 37°C. Cells were stained on the surface with CD3 and CD8 Abs as well as intracellularly with IFN-γ, TNF-α, and IL-17 Abs and then analyzed by polychromatic flow cytometry for the expression of cytokines in CD3+CD8− (referred as CD4+ T-cells) cells. Shown is (A) the gating strategy for CD4+ T-cells identification and (B) representative dot plots of IFN-γ, TNF-α, and IL-17 production by HIV-specific and C. albicans-specific CD4+ T-cells. (C) Shown is the intracellular expression of cytokines by CFSElowCD4+ T-cells specific for SEB, CMV, C. albicans, and different HIV peptide pools in five different SP subjects. (TIF) [file pone.0032964.s003.tif]
