# Supplementary material for: The Colocalization Potential of HIV-Specific CD8+ and CD4+ T-Cells is Mediated by Integrin β7 but Not CCR6 and Regulated by Retinoic Acid
Source: PLoS One. 2012 Mar 28;7(3):e32964. doi: 10.1371/journal.pone.0032964 (PMC3314661; doi:10.1371/journal.pone.0032964)
Supplement: Table S1 — Screening for HIV-1 specific CD4+ T-cells responses using the CD154 co-culture assay. To identify antigen-specific CD4+ T-cells, the CD154/CD40L assay was performed as previously described (89). To this aim, PBMC from five HIV-infected SP subjects were stimulated with SEB (1 µg/ml), CMV-pp65 peptide pool (5 µg/ml), recombinant HIV-p24 protein (5 µg/ml), or HIV peptide pools (10 µg/ml) in the presence of anti-CD154-PE/Cy5 Abs (20 µl/well) and monensin (2 µM) for 16 hrs at 37°C. Cells were then harvested, stained for surface markers with fluorescence-conjugated Abs against CD3, CD4, integrin β7, CCR6, CXCR3, and CCR4, and analyzed by flow cytometry for the expression of homing markers on CD3+CD4+CD154+ T-cells. Results are expressed as % of CD4+CD154+ T-cells. Values included in the table were >1.5-fold higher than the background CD154 expression observed for cells cultured in the absence of antigen. (DOC) [file pone.0032964.s004.doc]

**Table S1:** Screening for HIV-1 specific CD4^+^ T-cells responses using the CD154 co-culture assay

|  | **% CD154^+^CD4^+^ T-cells** | | | | |
| --- | --- | --- | --- | --- | --- |
| **Subjects** | **SP 005** | **SP 007** | **SP 008** | **SP 011** | **SP 015** |
| Medium^#^ | 0.1 | 0.28 | 0.1 | 0.03 | 0.1 |
| SEB | 3.49^&^ | 7.38 | 3.49 | 8 | 2.85 |
| CMV-pp65 | -^#^ | 0.46 | 1.12 | - | 0.89 |
| HIV-p24 | - | 0.42 | - | 0,08 | 1.28 |
| Nef _5139-5187_ | - | - | - | 0,09 | 0.52 |
| Nef _5139-5163_ | - | - | - | - | 0.61 |
| Nef _5164-5187_ | 0.33 | 0.58 | 0.33 | 0,08 | - |
| Gag _705-827_ | - | - | - | 0,08 | - |
| Gag _705-728_ | - | - | - | - | - |
| Gag _729-752_ | - | - | - | - | - |
| Gag _753-776_ | - | - | - | - | - |
| Gag _777-800_ | - | - | - | 0.07 | - |
| Gag _801-827_ | - | - | - | - | - |
| Pol _461-709_ | - | - | - | - | - |
| Pol _461-484_ | - | - | 0.29 | 0.09 | 0.28 |
| Pol _485-508_ | 0.29 | - | - | 0.27 | 0.79 |
| Pol _509-532_ | - | - | - | - | - |
| Pol _533-556_ | - | - | 0.15 | - | - |
| Pol _557-580_ | - | - | 0.17 | - | 0.9 |
| Pol _581-604_ | - | 0.48 | - | 0.09 | 2.34 |
| Pol _605-628_ | - | - | - | - | 0.21 |
| Pol _629-652_ | - | - | - | - | - |
| Pol _653-674_ | - | - | - | - | - |
| Pol _675-698_ | - | - | - | 0.06 | 0.57 |

^#^, % CD154^+^CD4^+^ T-cells; ^&^, CD154 expression was considered positive when the % of CD154^+^ T-cells in antigen-stimulated compared to the background was >1.5-fold higher.
